# Supplementary material for: Structural bioinformatic study of six human olfactory receptors and their AlphaFold3 predicted water-soluble QTY variants and OR1A2 with an odorant octanoate and TAAR9 with spermidine
Source: QRB Discov. 2024 Dec 9;6:e2. doi: 10.1017/qrd.2024.18 (PMC11811853; doi:10.1017/qrd.2024.18)

**Supplementary Information**

**Structural bioinformatic study of six human olfactory receptors and their AlphaFold3 predicted water-soluble QTY variants and OR1A2 with an odorant**

Finn Johnsson^1^, Taner Karagöl^2,¶^ , Alper Karagöl^2,¶^ , Shuguang Zhang^3,*^

^1^St Paul’s School, London, Lonsdale Road, London, SW13 9JT, UK

^2^Istanbul University Istanbul Medical Faculty, Istanbul, Turkey

^3^Laboratory of Molecular Architecture, Media Lab, Massachusetts Institute of Technology, 77 Massachusetts Avenue, Cambridge, MA, 02139, USA

^¶^These authors contribute equally.

*To whom the correspondence should be addressed.

Email:

Finn Johnsson, [finn.johnsson@icloud.com](mailto:finn.johnsson@icloud.com) ORCID: [0009-0009-9132-9488](https://orcid.org/0009-0009-9132-9488)

Taner Karagöl, [taner.karagol@gmail.com](mailto:taner.karagol@gmail.com) ORCID: [0009-0005-1011-7661](https://orcid.org/0009-0005-1011-7661)

Alper Karagöl, [alper.karagol@gmail.com](mailto:alper.karagol@gmail.com) ORCID: [0009-0001-7864-0732](https://orcid.org/0009-0001-7864-0732)

Shuguang Zhang, [Shuguang@MIT.EDU](mailto:Shuguang@MIT.EDU) ORCID: [0000-0002-3856-3752](https://orcid.org/0000-0002-3856-3752)

**Table S1. SwissParam scores of octanoic acid - receptor complexes.**

| **Receptor** | **SwissParam Score^1^** |
| --- | --- |
| **OR52cs** | -6.1091 |
| **OR1A1** | -6.0243 |
| **OR1A2** | -6.1848 |
| **OR51E1** | -6.0030 |
| **OR51E2** | -5.7291 |

^1^SwissParam scores were computed by SwissDock web server. Best scores for each complex were listed.


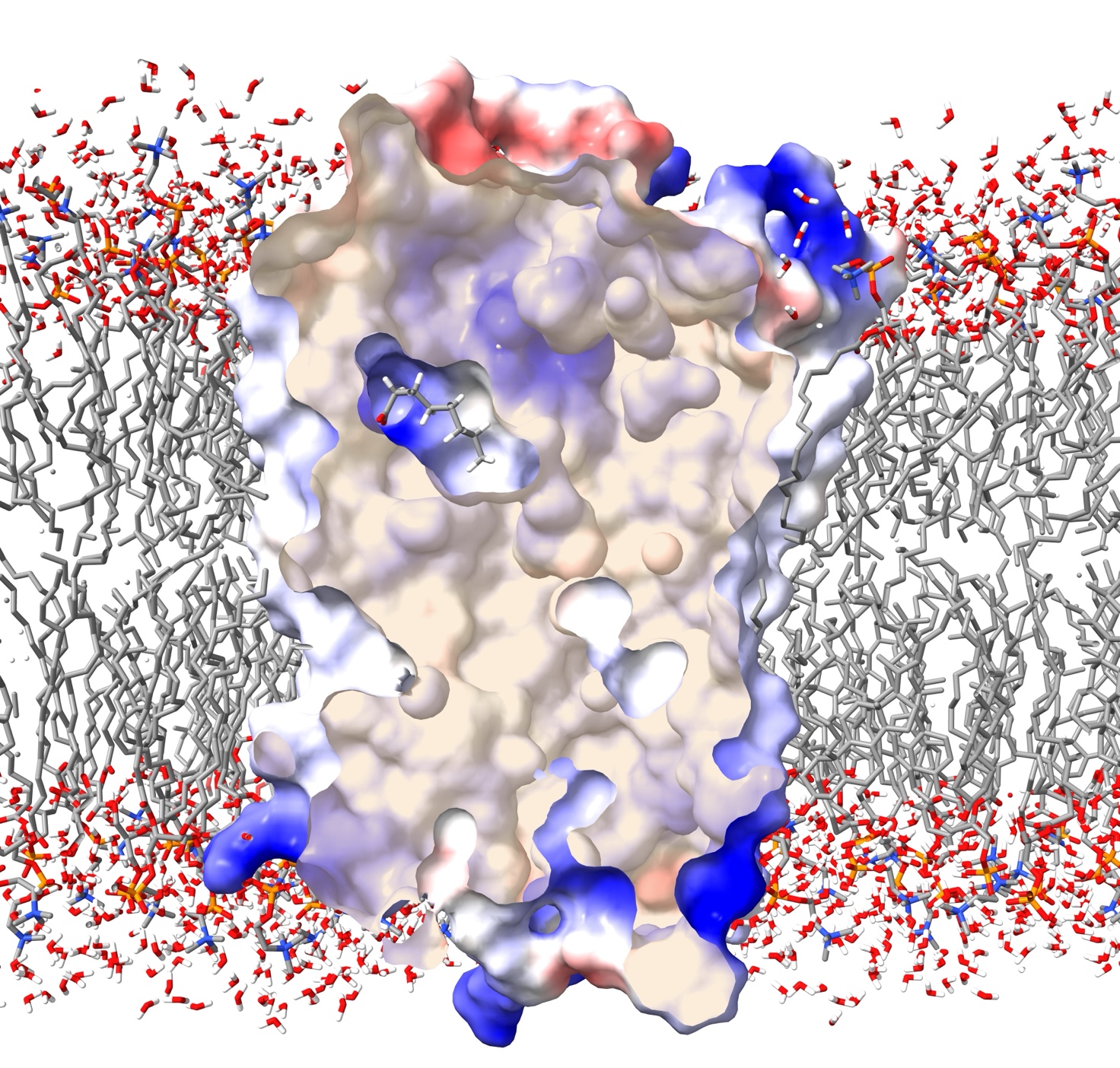


**Figure S1. OR1A2-octanoic acid complex in the membrane environment.** Octanoic acid (OCA) is positioned within a hydrophobic cleft. The electrostatic potential surface highlights the compatibility of the binding pocket for the hydrophobic tail and polar head group of OCA.


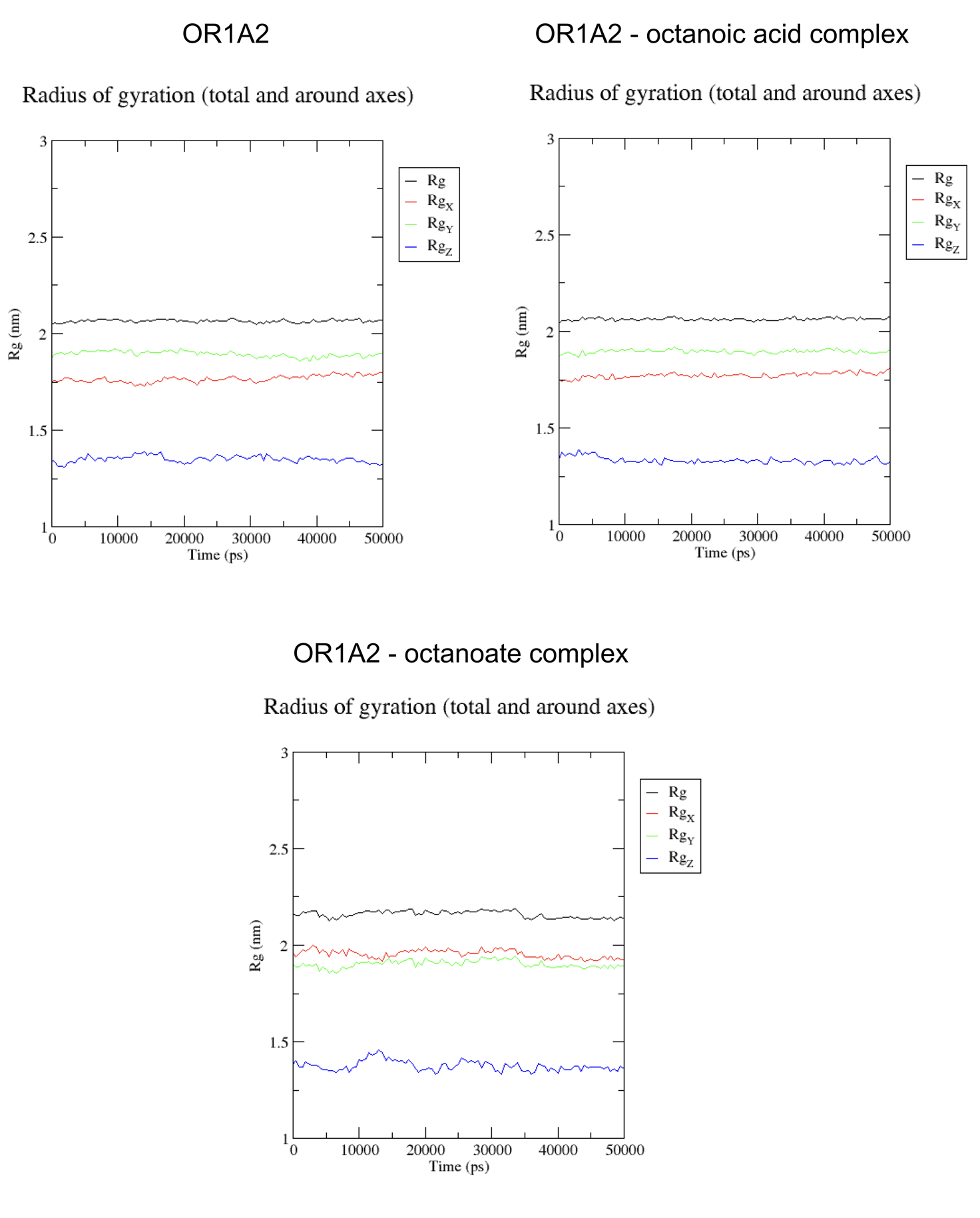


**Figure S2. Radius of gyration during the 50ns MD simulation.**


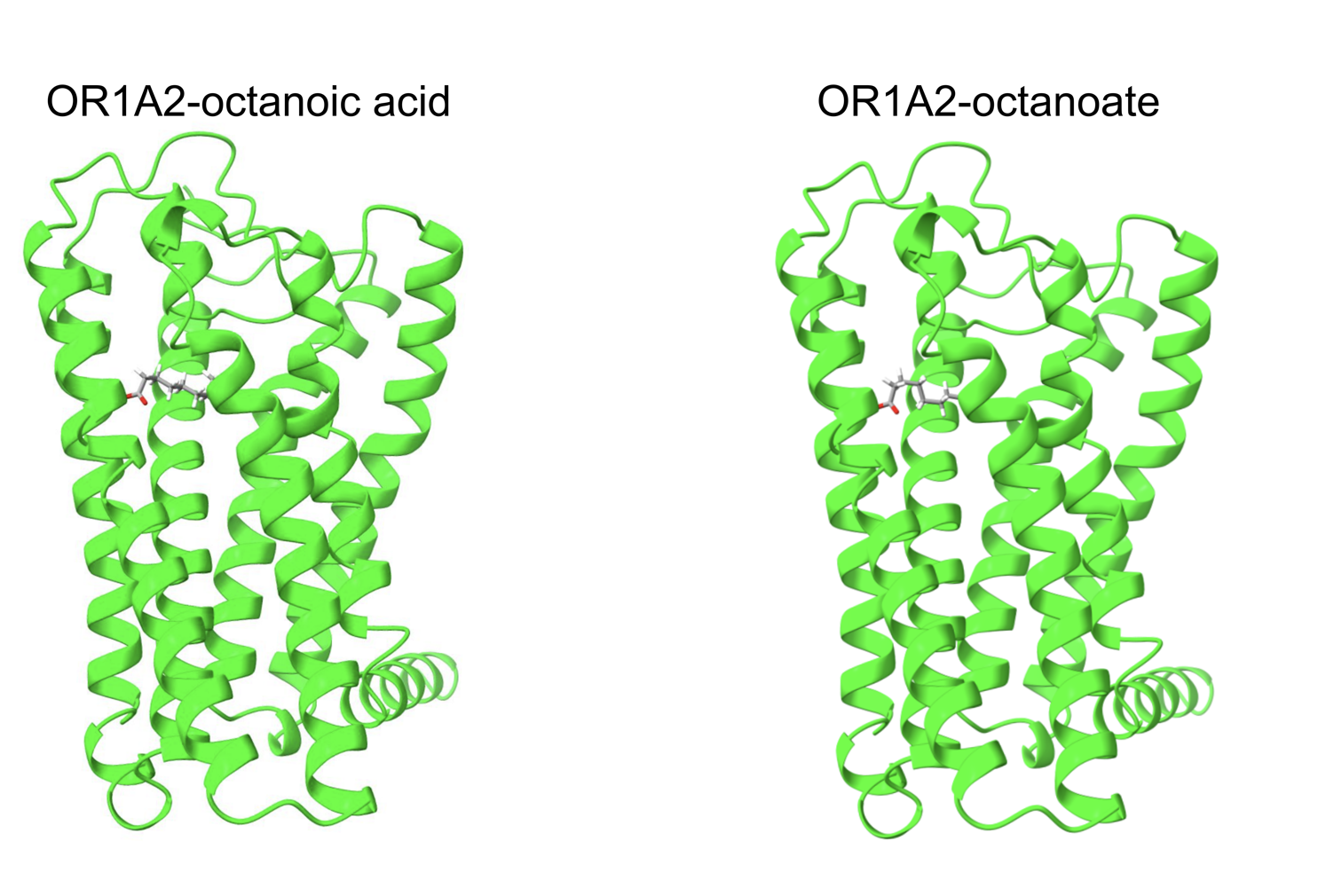


**Figure S3. OR1A2 in complex with octanoic acid (left) and octanoate (right).** Structural representation of the OR1A2 complex bound with octanoic acid (eft) and octanoate (right). The AlphaFold predicted protein structure in green cartoon representation, with the ligand octanoic acid shown as a stick model.


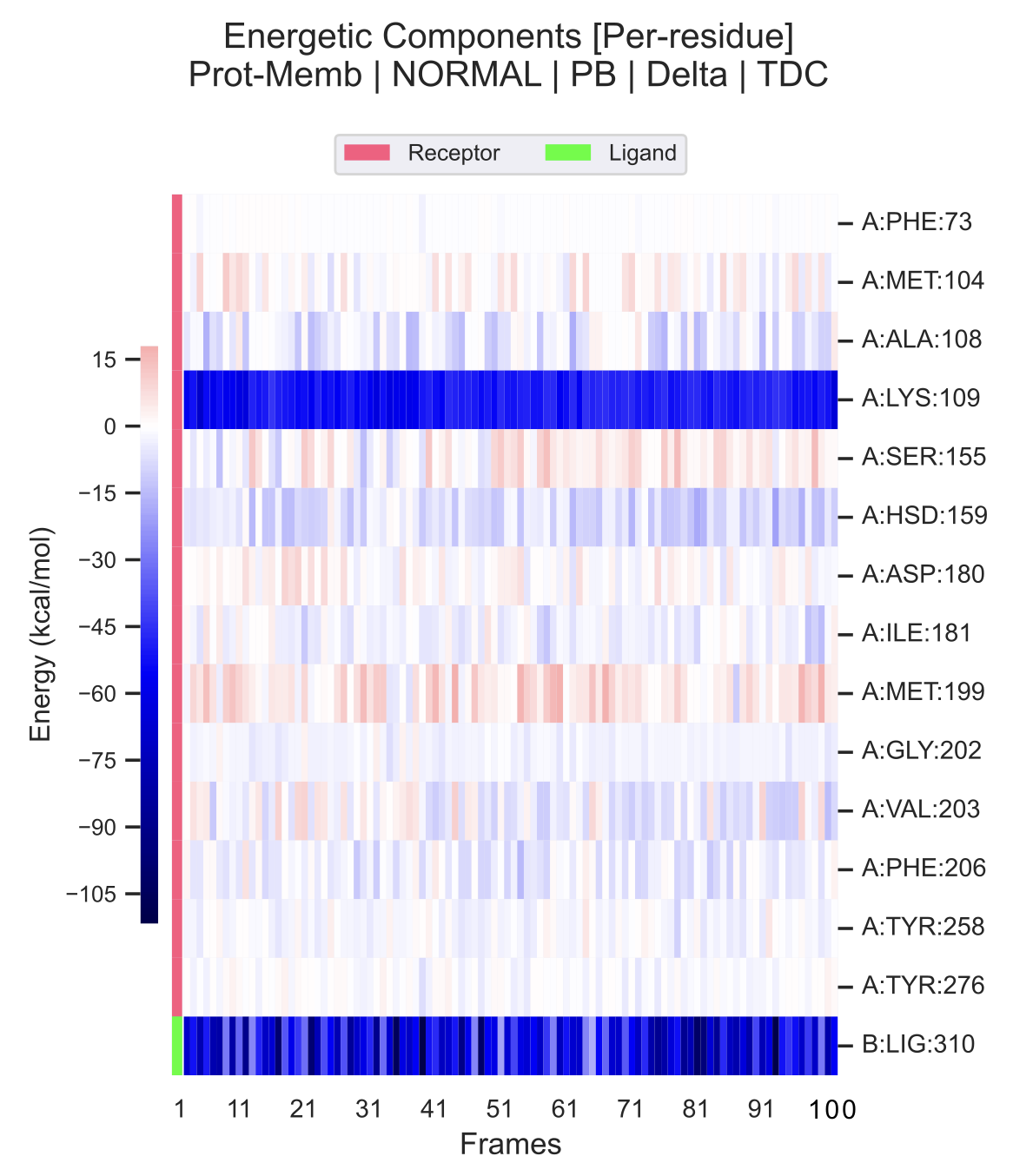


**Figure S4. Residue decomposition analysis of OR1A2-octanoate complex during 50ns MD simulation.** Heatmap of per-residue energetic contributions to octanoate binding (B:LIG) across 100 simulation frames (50ns). The heatmap shows the contributions of individual residues (on the y-axis) to the binding energy (indicated by the color scale, red to blue).


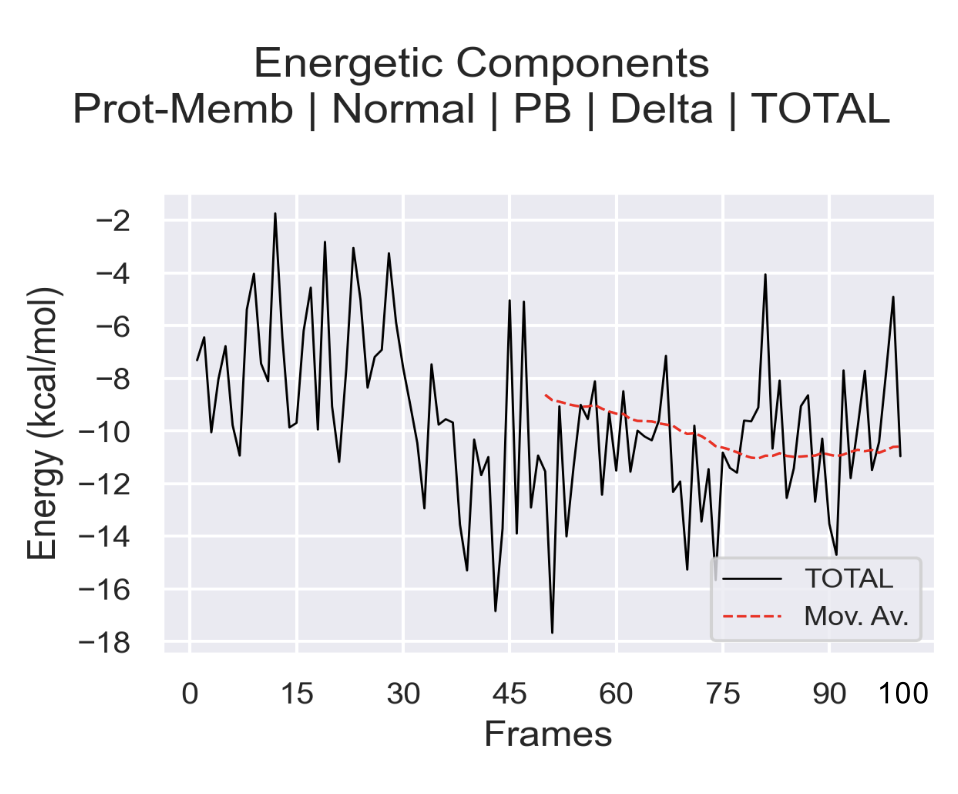


**Figure S5. MMPSA calculated binding free energy of OR1A2-octanoate complex during 50ns MD simulation.** MMPBSA calculated total binding energy of octanoate to the OR1A2 over 100 simulation frames (50ns). The black line represents the binding energy, while the red dashed line indicates the moving average trend across the frames.

**Figure S6a-f. For clarity and easy visualization, the enlarged protein sequence alignments of six native olfactory receptor proteins with their water-soluble QTY variants**. The symbols | and * indicate whether amino acids are identical or different, respectively. Note the Q, T, and Y amino acids replacing L, V and I, and F, respectively. The alpha helices (blue) are shown above the protein sequences. The characteristics of natural and QTY variants listed are isoelectric focusing (pI), molecular weight (MW), total variation %, and transmembrane variation %. The alignments are: a) OR51E2 *vs* OR51E2^QTY^, b) OR52cs *vs* OR52cs^QTY^, c) TAAR9 *vs* TAAR9^QTY^, d) OR51E1 *vs* OR51E1^QTY^, e) OR1A1 *vs* OR1A1^QTY^, and f) OR1A2 *vs* OR1A2^QTY^.

**a) OR51E2 *vs* OR51E2^QTY^**


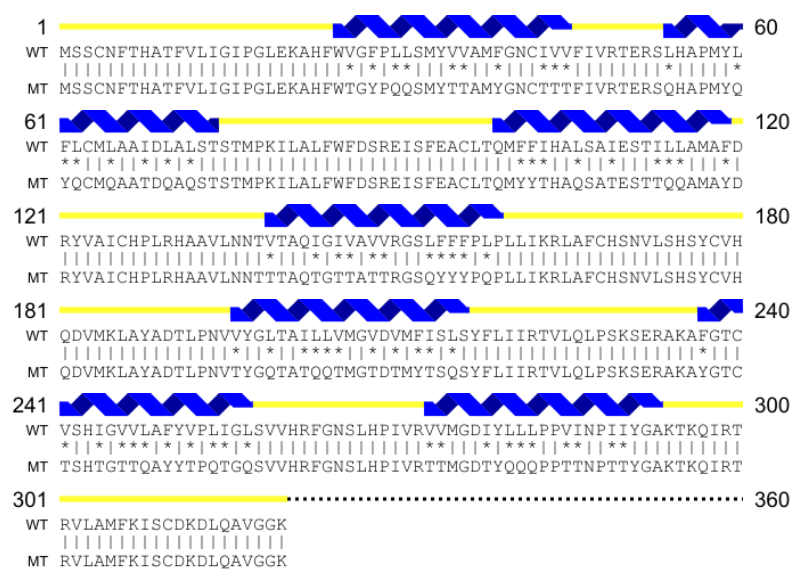


**b) OR52cs *vs* OR52cs^QTY^**


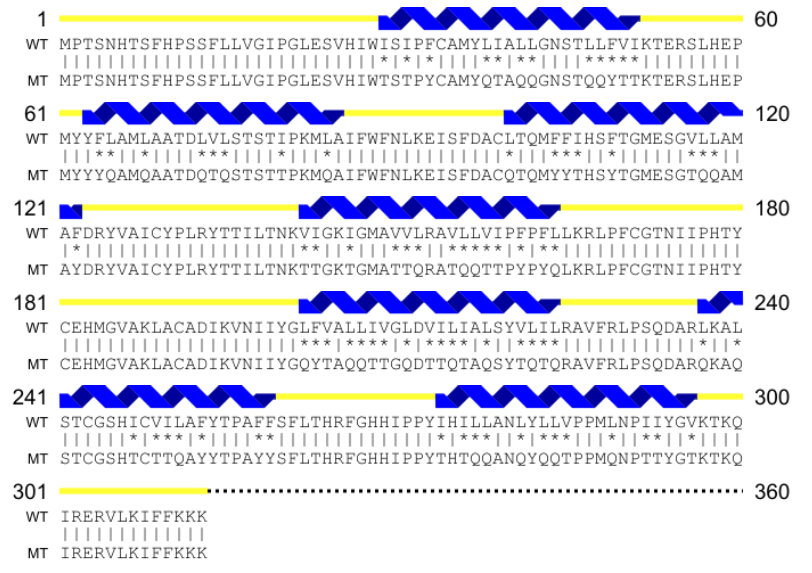


**c) TAAR9 *vs* TAAR9^QTY^**


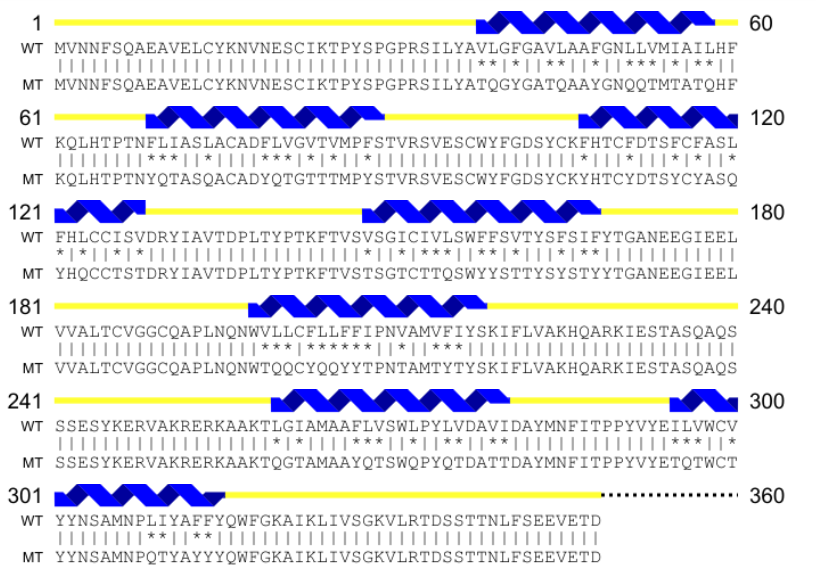


**d) OR51E1 *vs* OR51E1^QTY^**


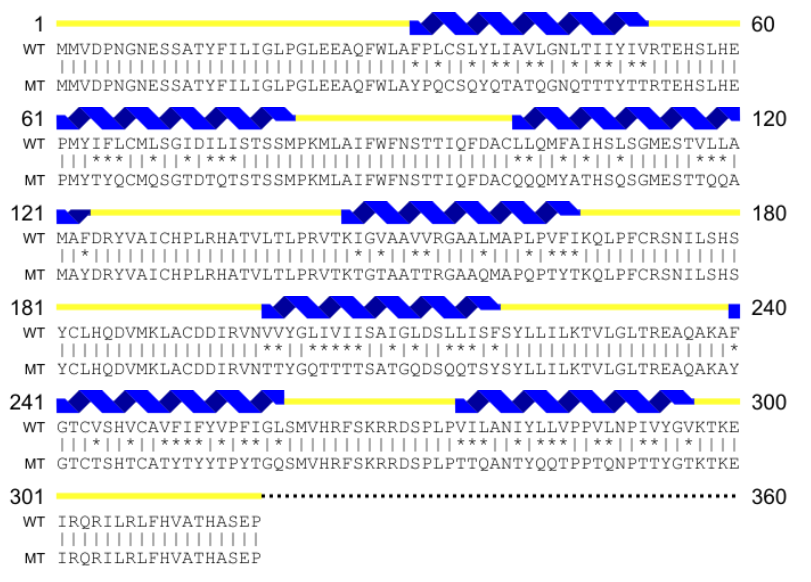


**e) OR1A1 *vs* OR1A1^QTY^**


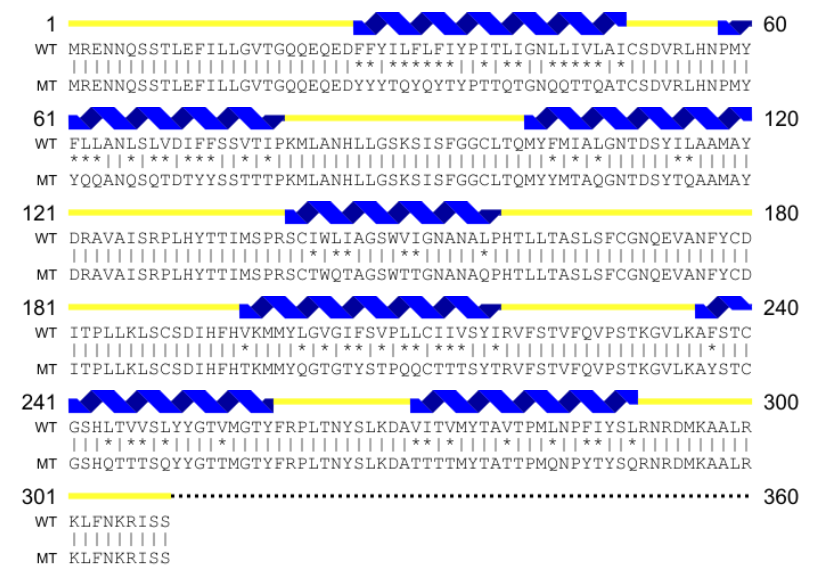


**f) OR1A2 *vs* OR1A2^QTY^**


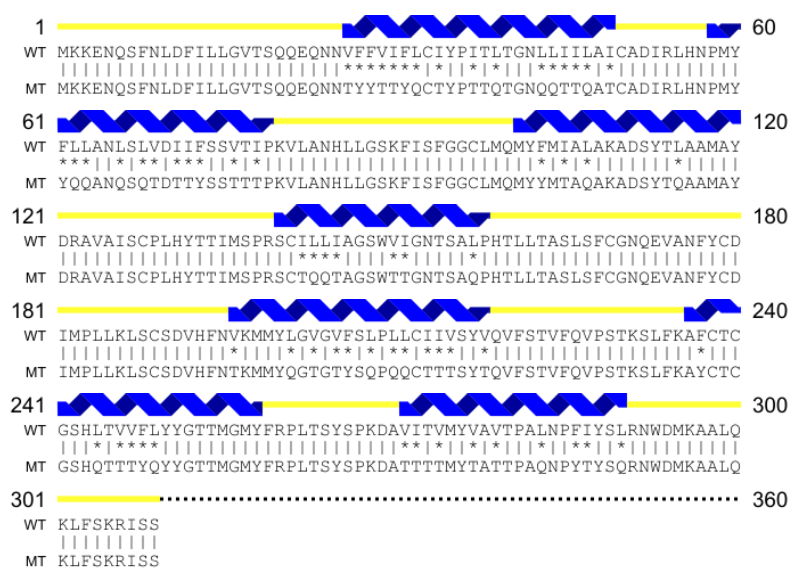

Supplement: Johnsson et al. supplementary material [file S2633289224000188sup001.docx]
